# Food Intake and Dietary Glycaemic Index in Free-Living Adults with and without Type 2 Diabetes Mellitus

**DOI:** 10.3390/nu3060683

**Published:** 2011-06-09

**Authors:** Susan C. McGeoch, Grietje Holtrop, Claire Fyfe, Gerald E. Lobley, Donald W. M. Pearson, Prakash Abraham, Ian L. Megson, Sandra M. MacRury, Alexandra M. Johnstone

**Affiliations:** 1 Department of Diabetes, Aberdeen Royal Infirmary, Aberdeen AB25 2ZN, UK; Email: donald.pearson@nhs.net (D.W.M.P.); p.abraham@nhs.net (P.A.); 2 Biomathematics and Statistics Scotland, Edinburgh EH9 3JZ, UK; Email: g.holtrop@abdn.ac.uk; 3 The Rowett Institute of Nutrition and Health, University of Aberdeen, Aberdeen AB21 9SB, UK; Email: c.fyfe@abdn.ac.uk (C.F.); g.lobley@abdn.ac.uk (G.E.L.); alex.johnstone@abdn.ac.uk (A.M.J.); 4 Department of Diabetes & Cardiovascular Science, University of Highlands and Islands, Inverness IV2 3JH, UK; Email: ian.megson@uhi.ac.uk (I.L.M.); sandra.macrury@uhi.ac.uk (S.M.M.)

**Keywords:** type 2 diabetes mellitus, diet, glycaemic index, lifestyle, food intake

## Abstract

A recent Cochrane review concluded that low glycaemic index (GI) diets are beneficial in glycaemic control for patients with type 2 diabetes mellitus (T2DM). There are limited UK data regarding the dietary GI in free-living adults with and without T2DM. We measured the energy and macronutrient intake and the dietary GI in a group (*n* = 19) of individuals with diet controlled T2DM and a group (*n* = 19) without diabetes, matched for age, BMI and gender. Subjects completed a three-day weighed dietary record. Patients with T2DM consumed more daily portions of wholegrains (2.3 *vs.* 1.1, *P* = 0.003), more dietary fibre (32.1 *vs.* 20.9 g, *P* < 0.001) and had a lower diet GI (53.5 *vs.* 57.7, *P* = 0.009) than subjects without T2DM. Both groups had elevated fat and salt intake and low fruit and vegetable intake, relative to current UK recommendations. Conclusions: Patients with T2DM may already consume a lower GI diet than the general population but further efforts are needed to reduce dietary GI and achieve other nutrient targets.

## 1. Introduction

In the UK, lifestyle intervention is the first line of management in type 2 diabetes mellitus (T2DM) [1]. Both the NICE [1] and ADA [2] guidelines recommend that a dietary plan for patients with T2DM should follow the principles of healthy eating. This includes carbohydrate from fruits, vegetables, wholegrains, and pulses (thus a high fibre and lower glycaemic index (GI) diet), reduction in salt intake, the inclusion of low-fat milk and oily fish, and reduced saturated and trans fatty acid intake (e.g., as described by the Food Standards Agency [3], for healthy subjects and by Diabetes UK [4], for patients with type 2 diabetes). This dietary approach is supported by a recent Cochrane review [5] based on meta-analysis of dietary intervention studies. It concluded that low GI diets were beneficial in T2DM and could lead to improvement in glycaemic control with a 0.5% reduction in HbA1c, used as a marker of glucose excursion. Improved glycaemic control through diet can minimise medication, lessen risk of diabetic complications, improve quality of life and increase life expectancy [5]. Nonetheless, there are limited data available on the dietary GI in free-living patients with and without T2DM. Therefore it is unknown if the GI diets in patients differ from those habitually adopted by individuals with no disease or, indeed, if patients’ diets meet current healthy eating advice and nutritional guidelines. The dietary GI of free-living patients with diet-managed T2DM was assessed by Barclay *et al.* [6], using a food frequency questionnaire (FFQ) in Australians with and without T2DM. They report a similar average GI of 55 in the patients and 57 for those without T2DM. The use of a FFQ is questionable however, because of errors associated with reliance on volunteer memory and variation in individual interpretation of food portion size and frequency. There are no comparable data from a UK cohort of free-living patients. The “gold standard” of assessing food, energy and nutrient intake is considered to be the weighed dietary record [7]. Furthermore, although there are many dietary intervention studies comparing low- and high-GI intervention diets in adults [8,9,10,11], there is no consensus as to reference ranges for defining “low” and “high” GI. The reported intervention studies (that assess impact on glycaemic control) involved a dietary range from 43 to 93 GI units in adults.

In view of the current paucity of information in this area, we sought to determine the dietary macronutrient composition and GI in a group of free-living individuals with diet-managed T2DM and a group of controls matched for age, BMI and gender. In addition, their nutrient intake was compared with current UK dietary recommendations and dietary reference values for healthy eating.

## 2. Methods

### 2.1. Subject Characteristics

All volunteers were recruited for studies undertaken by the Human Nutrition Unit at the Rowett Institute of Nutrition and Health, University of Aberdeen. Nineteen patients with T2DM managed by diet alone were recruited by newspaper advertisement and from General Practice. The diagnosis of T2DM was based on standard WHO criteria [12]. Exclusion criteria included medical co-morbidities such as cancer, gastrointestinal disease or mental health issues, based on a medical screening questionnaire. Subjects with no apparent T2DM were recruited in a similar manner and underwent a fasting blood glucose measurement to confirm the absence of diabetes. Volunteers with T2DM had received standard National Health Service dietary advice to follow a healthy eating plan, based on Diabetes UK literature [4]. The North of Scotland Research Ethics Service approved the study. Written informed consent was obtained from all participants.

### 2.2. Food Intake Recording

Volunteers with type 2 diabetes recorded all food and drinks consumed for three consecutive days. Volunteers without diabetes recorded food and drink intake for the same three days of the week. Volunteers were provided with digital electronic scales with a tare facility and a food diary for recording food and drinks consumed, time of consumption, weight of food and drink, cooking method and any leftovers. They were instructed to record all recipes and to keep packaging for ready-to-eat food products.

### 2.3. Analysis of Food Intake Data

Diets were analysed using WinDiets Nutritional Analysis Software Suite (Version 1.0, The Robert Gordon University, Aberdeen, UK), a computerized version of McCance and Widdowson: The Composition of Foods 6th Edition [13]. Standard portion sizes were used to input foods with household measures or with missing weights or portion sizes [14]. Total energy and nutrient intake for each meal were quantified. The GI of the different foodstuffs was taken from published reference tables [15]. The tables have some information on ready meals (e.g., pizza, sushi). When this information was not available, the constituent ingredients were entered individually (e.g., spaghetti & tomato sauce for spaghetti bolognase).

- Dietary GI was calculated as: The sum of the GI value of each foodstuff multiplied by available carbohydrate content (total carbohydrate minus dietary fibre) expressed as a proportion of the total available carbohydrate for the day;
- Dietary glycaemic load (GL) was calculated as: The sum of the product of the GI for each foodstuff and its available carbohydrate content, divided by 100.

The number of serving of fruit and vegetables was determined as per the British Nutrition Foundation guidelines [16] and number of servings of wholegrain foods was estimated using the US Department of Health guidelines [17]. 

### 2.4. Estimation of Basal Metabolic Rate (BMR)

BMR for all the volunteers was calculated as described by Schofield *et al.* [18]. The energy intake (EI)/BMR ratio for each subject was then calculated.

### 2.5. Comparison with a Three Day Diet Based on Dietary Recommendations for Type 2 Diabetes

Based on the menu planner facility on the Diabetes UK [19], a menu was selected for a male aged 60 years, weight of 93 kg, with light activity for weight maintenance, based on our mean volunteer characteristics. The three-day menu selected was analysed using WinDiets Nutritional Analysis Software Suite. Total energy and nutrient content for each meal were quantified and the GI, GL and number of portions of fruit and vegetables and whole-grains were determined as above. Means of the values for the three days were then used for comparative purposes. A sample intake for one day included:

Breakfast: 44 g wholegrain breakfast cereal, 200 g semi skimmed milk, 150 g unsweetened fruit juice;
Lunch: 30 g Lettuce, 85 g tomato, 15 g reduced calorie mayonnaise, 175 g cooked chicken breast, 125 g low fat fruit yoghurt, 140 g wholemeal bread;
Dinner: 175 g cooked extra lean minced beef, 150 g boiled white rice, 200 g cauliflower, 170 g fresh pears, 60 g vanilla ice-cream;
Snacks: 200 g semi skimmed milk, 34 g cereal bar, 100 g red apples, 25 g raisins.

### 2.6. Statistical Analysis

For statistical analysis, volunteers with diabetes were paired with 19 volunteers without diabetes on the basis of age, sex, BMI and EI/BMR ratio. Only days where the food diary record was complete were used. For each pairing, food diary data for the same three days of the week were used. Data were analyzed by paired *t*-test. Residual plots were inspected for normality and constant variance. If these assumptions were violated a nonparametric test (Wilcoxon matched pairs test) was used instead. This was necessary for alcohol, fibre intake and portions of fruit, vegetables and whole-grains. Data were analysed using GenStat for Windows (11th Edition); VSN International, Hemel Hempstead, UK.

## 3. Results

### 3.1. Comparison of Groups with and without Type 2 Diabetes

Volunteer characteristics are shown in Table 1.

**Table 1:** Volunteer characteristics Mean (SD).

	Volunteers without diabetes *n* = 19	Volunteers with type 2 diabetes *n* = 19	*p*
**Age (years)**	57.8 (6.5)	60.1 (7.2)	0.41
**Male:Female**	15:4	15:4	-
**BMI (kg/m^2^)**	32.1 (6.4)	32.5 (5.4)	0.86
**BMR (kJ/day)**	7713 (1289)	7625 (1186)	0.67
**Energy intake/BMR ratio (kJ)**	1.21 (0.24)	1.26 (0.43)	0.67
**HbA1c (%)**	-	6.9 (Range: 5.8–8.9)	-

There were no significant differences for age, BMI, BMR and male:female ratio between the two groups. The mean BMI of both groups falls within the obese range. The mean EI/BMR was also similar at 1.21 for volunteers without T2DM and 1.26 for volunteers with T2DM. Both values are below the cut-off described by Goldberg *et al.* [20] who reported that levels of EI below 1.35 × BMR are incompatible with long term maintenance of energy balance and suggests that both groups had a similar tendency to under-report food intake or may simply reflect a modern day sedentary lifestyle.

Table 2 shows total energy, macronutrient intake and dietary GI and GL for the two groups. 

**Table 2:** Daily total energy, macronutrient intake and dietary GI and GL for both groups.

	Volunteerswithout diabetes ( *n* = 19)	Volunteers with type 2 diabetes ( *n* = 19)	Devised DUK diet for type 2 diabetes	*P* (comparison between volunteers with and without diabetes)
**Energy (kJ)**	9289.9 (2331.4) 6087–15147	9527.9 (3350.4) 3936–15478	9427.4	0.79
**Energy (kcal)**	2222.5 (557.8) 1456–3624	2270.5 (812.2) 942–3703	2255.4	0.82
**Fat % of energy**	32.1 (8.1) 15–43	33.5 (8.3) 22–54	26.8 *	0.59
**Protein % of energy**	17.2 (3.9) 11–26	20.3 (3.5) 14–29	27.9 ^$^	**0.034**
**CHO % of energy**	46.0 (9.2) 32–64	47.2 (10.4) 28–68	45.4	0.72
**Saturated fat % of energy**	11.7 (4.2) 3.7–18.9	10.8 (4.3) 0.7–21	8.6 *	0.51
**Salt (g)**	8.2 (2.9) 2.6–14.5	8.8 (3.6) 3.2–15.9	5 ^$^	0.57
**Alcohol (kJ)**	571.3 (767.2) 0–2551	0.14 (0.6) 0–2.65	0	**<0.001**
**Saturated fat (g)**	30.0 (19.9) 7.5–77.5	31.4 (19.9) 1.1–87.5	21.9	0.81
**Total sugars (g)**	96.2 (50.3) 24.7–235	99.2 (36.2) 47–179	130.2 *	0.84
**Dietary fibre (g)**	20.9 (4.4) 10.7–20.9	32.1 (15.6) 11.0–76.9	29.2	**<0.001**
**Portions of fruit and vegetables**	3.81 (2.5) 0–8.7	3.94 (1.7) 2.0–8.3	6.3 ^$^	0.93
**Portions of wholegrain foodstuffs**	1.1 (0.84) 0–3.0	2.3 (1.5) 0–6.0	2	**0.003**
**Available CHO (g)**	241.3 (62.5) 152–361	242.8 (83.9) 108–403	237.6	0.95
**Dietary GI**	57.7 (4.8) 47–65	53.5 (3.9) 47–61	54.4	**0.009**
**Dietary GL**	139.9 (40.2) 81–223	130.6 (48.0) 59–220	129.3	0.49

Mean (SD) Range; Comparison between the diets of volunteers with T2DM and Diabetes UK menu: * *P* < 0.05, ^$^ *P* < 0.001; GI: Glycameic Index, GL: Glycaemic Load.

Total EI and percentage of energy derived from either fat or carbohydrate were similar for the two groups, with patients with T2DM consuming a higher percentage of energy from protein (20.3 *vs.* 17.2%, *P* = 0.034). The group with T2DM also ate more dietary fibre (32.1 *vs.* 20.9 g, *P* < 0.001) explained in part by consumption of more portions of whole-grains (2.3 *vs.* 1.1, *P* = 0.003). These differences contributed to the lower dietary GI (53.5 *vs.* 57.7, *P* = 0.009) for this group, although the dietary GL was similar (*P* = 0.49), as were the sugar (*P* = 0.84) and available carbohydrate (*P* = 0.95) intakes.

### 3.2. Comparison of Group Data with Reference Values

Table 2 also details the results for total energy intake, macronutrient composition, GI and GL calculated from the Diabetes UK (DUK) meal plan for patients with T2DM. If this planner is considered a guidance level, then several of the nutrient intakes were met, including dietary GI, GL, fibre intake and portions of whole-grains. In contrast, intake of salt was above the 5 g/day recommendation (at 8.8 g/day), while daily fruit and vegetable consumption (average 3.9 portions) was well below the 6.3 level from the DUK meal plan. Saturated fat intake was also much higher at 31.4% in comparison to the healthier 21.9% in the planner; total fat intake at 33.5% was also higher than the DUK meal plan (26.8%). The percentage of total energy derived from protein, however, was less than the DUK meal plan (20.3 *vs.* 27.9%).

Notably, both patients with T2DM and subjects without disease had similar levels in total fat and saturated fat intake, expressed as a percentage of total energy intake (Table 2). However, these levels would only be considered acceptable for a non-diseased population, as they are only slightly higher than that recommended for the general population (healthy, non diabetic) for optimal health (DOH [21]: 30% fat and 10% saturated fat from energy). Both groups had low fruit and vegetable intake, much less than the 5–6 portions a day recommended for the UK population while salt intake was higher than that recommended for the general population (6 g/day [3]). Thus, these trends are not specific to the patient group with T2DM, but reflect more the general problems associated with the typical “Scottish Diet”.

## 4. Discussion

To our knowledge this is the first study to evaluate the dietary GI for a group of free-living diet-managed individuals with T2DM in the UK. This study therefore provides an estimation of habitual intake that provides a reference for dietary intervention studies performed under controlled laboratory conditions (with test meals and novel products).

### 4.1. Dietary Glycaemic Index (GI)

The present study indicated that patients with T2DM consumed a relatively healthy diet, with more portions of whole-grains, dietary fibre and a slightly lower dietary GI than a matched group of obese individuals with no T2DM. These findings probably reflect the dietary advice received at diagnosis. Indeed, the meal plan from DUK had a similar GI of 54. These findings are also comparable with those of Barclay *et al.* [6], using the FFQ tool, who reported a dietary GI of 55 and 57 for subjects with and without T2DM, respectively. Other cultures report a slightly higher dietary GI in healthy subjects, e.g., one study of Japanese women involved a mean GI of 64 [22]. 

There are numerous intervention studies comparing high and low GI diets in the management of T2DM and these are summarised in Table 3 [8,9,10,23,24,25,26,27,28].

**Table 3:** The glycaemic index of the diets used in studies that showed an improvement in glycaemic control with a low GI diet.

Study	High GI diet mean GI	Low GI diet mean GI	Difference in GI
Wolever *et al.* [24]	87	60	27
Jenkins *et al.* [23]	90.5	67.3	23.2
Frost *et al.* [10]	82	77	5
Fontvieille *et al.* [9]	64	38	26
Jarvi *et al.* [25]	82.7	56	26.7
Jenkins *et al.* [26]	86	62	24
Rizkalla *et al.* [27]	71	39	32
Jimenez-Cruz *et al.* [28]	56	44	12
Brand *et al.* [8]	91	77	14
**Mean**	**78.9**	**57.8**	**21.1**

The arithmetic mean of the low GI diets was 58 (range 38–77) while the mean of high GI diets was 79 (range 56–91). Compared with these intervention studies, our current volunteers are therefore already eating a diet within the “low” GI range. Within the confines of normal “healthy” eating, the options to reduce the dietary GI much further are probably limited and, therefore, additional dietary advice to lower the dietary GI may not lead to further improvements in glycaemic control. Interestingly, the volunteers without T2DM had only slightly higher dietary GI, although other aspects of their diet were less healthy. The usefulness of the concept of GI has been questioned in relation to healthy eating advice, in particular for subjects without diabetes as it is subject to technical [29], practical [30] and theoretical limitations [31]. Furthermore, people do not usually eat single foods and combining carbohydrate products with other macronutrients can affect the overall estimation of meal GI [29]. Nonetheless, the concept of GI is relatively easily understood by patients, and although flawed, is probably the most sensible approach currently available. 

### 4.2. Total Energy Intake

Mean EI was similar for both the groups, as was the average EI/BMR ratio. Similar values have been reported in other patient studies [6,32] and healthy subjects [33]. It is recognised that dietary assessment methods have a strong bias towards underestimation of habitual energy intake [34], but weighed diet records are considered the “gold standard” when examining free-living energy and nutrient intake [7].

### 4.3. Diet Composition

The group with T2DM consumed similar proportions of total energy as carbohydrate and fat but with a higher fraction from protein compared with the non-patient group. These proportions of intake are also comparable with other work based on food diary data [6,35,36]. Fibre intake in both groups exceeds current UK guidelines for the general population (18 g/day) [21] and is higher than the average fibre intake in the general Scottish population (12 g/day, [37]), suggesting that both groups were more nutritionally aware than the general public. Both groups, however, consumed in excess of the recommended daily salt intake. In view of the increased cardiovascular risk associated with diabetes [38] and the link between salt intake and hypertension [39] this is identified as an area where further education may improve diet and health profiles.

### 4.4. Comparison with the DUK Menu Plan

In comparison with the menu plan from DUK, patients met the targets for GI, GL, dietary fibre and whole-grains. The number of portions of fruit and vegetable intake was low, but similar to that reported for the general Scottish population (3.2 for women and 3.0 for men [40]). The protein content of the DUK menu (1.7 g/kg body weight) is higher than that usually recommended for those with diabetes where 1 g/kg body weight should not be exceeded [41].

### 4.5. Implications for Clinical Practice

It is reassuring that volunteers were able to achieve dietary GI comparable with those used in dietary intervention studies that have resulted in an improved glycaemic control. This reinforces the benefits of dietary intervention as the first line management in type 2 diabetes. Whether even lower GI diets might be more beneficial cannot be ascertained from the current study, but there may be practical limitations for a habitual lifestyle. If the current results are typical of individuals with Type 2 diabetes stabilised by life-style advice, then current dietary advice seems to be effective in improving both fibre and whole grain consumption. Nonetheless, the data suggest that more education needs to be focused on reduction of salt and saturated fat intake plus a drive to further increase fruit and vegetable consumption.

It is also unclear whether low GI diets are the best dietary strategy for optimising glycaemic control in T2DM. Reduction in percentage of energy of the diet derived from carbohydrate will result in a lower dietary GL and it could be postulated that this might lead to improved glycaemic control. However, studies to date have not demonstrated convincing benefits of reduced carbohydrate diets on glycaemic control when compared with low GI diets or standard healthy eating. For example, Daly *et al.* [42] randomised volunteers to receive either education regarding standard healthy eating or a low carbohydrate diet and found that although participants who followed the low carbohydrate diet lost more weight and had improved lipid profiles there were no differences in glycaemic control between the two groups. Further research needs to be undertaken, therefore, to determine the best dietary strategies to optimise glycaemic control in individuals with T2DM.

### 4.6. Study Limitations

The subject numbers are relatively small and it is recognised that dietary assessment methods can be inaccurate and are biased towards patient under-reporting [7]. The determination of dietary GI and GL are from published reference tables that are often incomplete and may lead to under or over-estimation of meal values. These patient volunteers also had been effective in regulating their diabetes by lifestyle changes alone and therefore may not be typical of the attitude and/or determination of those newly diagnosed with the disease. As a cohort, they do illustrate the potential effectiveness of this type of disease management.

## 5. Conclusions

Patients with diet-managed type 2 diabetes mellitus may already consume a low-GI diet, but may not be meeting other nutrient targets, and dietary advice should also focus on reducing salt intake and increasing fruit and vegetable intake.
